# La sténose trachéale sévère post-intubation prolongée

**DOI:** 10.11604/pamj.2017.28.247.9353

**Published:** 2017-11-21

**Authors:** Samia Frioui, Faycel Khachnaoui

**Affiliations:** 1 Physical Medicine and Rehabilitation Department, Sahloul Hospital, Sousse, Tunisia

**Keywords:** Sténose trachéale, intubation, trachéotomie, chirurgie, Tracheal stenosis, intubation, tracheotomy, surgery

## Abstract

The rate of post-intubation tracheal stenosis (PITS) varies from 10 to 22% according to the studies. Only 1-2% of these stenoses are severe or symptomatic and manifest as inspiratory dyspnea that doesn't improve under corticosteroid treatment. STPI often occurs in patients with altered general status and this complicates their management. We report the case of a 43-year old hypertensive patient with a 1-year history of haemorrhagic stroke due to hypertensive peak, who had been in a coma for 3 months, requiring prolonged intubation and tracheotomy. Tracheal cannula removal had been proven time and again, but it was impossible due to respiratory distress. ENT exam showed important subglottic stenosis above the hole due to tracheotomy. Ct scan confirmed tracheal stenosis (A, B, C). Tracheoscopy under general anesthesia objectified subglottic stenosis at 1 cm from the glottic floor. The patient underwent surgery with proximal tracheal resection of 3cm, posterior cricoid mucosectomy and cricotracheal anastomosis. Postoperative outcome was favorable. The diagnosis of PITS is sometimes difficult, but it should be suspected in any patient with dyspnoea of recent onset or with unusual dyspnoea after intubation and/or tracheotomy. Tracheal resection and anastomosis, as they have been performed in our study, are the treatment of choice for tracheal stenosis.

## Image en médecine

La fréquence des sténoses trachéales post-intubation (STPI) varie selon les études de 10 à 22 %. Seulement 1 à 2% de ces sténoses sont sévères ou symptomatiques et se manifestent par un tableau de dyspnée inspiratoire ne cédant pas sous traitement corticoïde. Les STPI surviennent souvent chez des patients à l'état général altéré, ce qui complique leur prise en charge. Nous rapportons le cas d'un patient âgé de 43 ans, hypertendu, qui a présenté il y a un an un Accident Vasculaire Cérébral hémorragique suite à un pic hypertensif avec un coma ayant duré 3 mois nécessitant une intubation prolongée et une trachéotomie. Une décanulation s'est avérée impossible à maintes reprises, se soldant à chaque tentative de décanulation par une détresse respiratoire. L'examen ORL spécialisé avait révélé une sténose importante sous glottique au-dessus de l'orifice de trachéotomie. Le TDM a confirmé la sténose trachéale (A, B, C). Une trachéoscopie sous anesthésie générale avait mis en évidence une sténose sous glottique à un cm du plan glottique. Le patient a été opéré et a eu une résection trachéale proximale de trois cm avec une mucosectomie cricoïdienne postérieure et une anastomose crico-trachéale. L'évolution post-opératoire était favorable. Le diagnostic de STPI est parfois difficile mais il doit être évoqué chez tout patient aux antécédents d'intubation et/ou de trachéotomie présentant une dyspnée d'apparition récente ou inhabituelle. La résection-anastomose trachéale, telle qu'elle a été faite pour notre patient, reste le traitement de référence.

**Figure 1:**
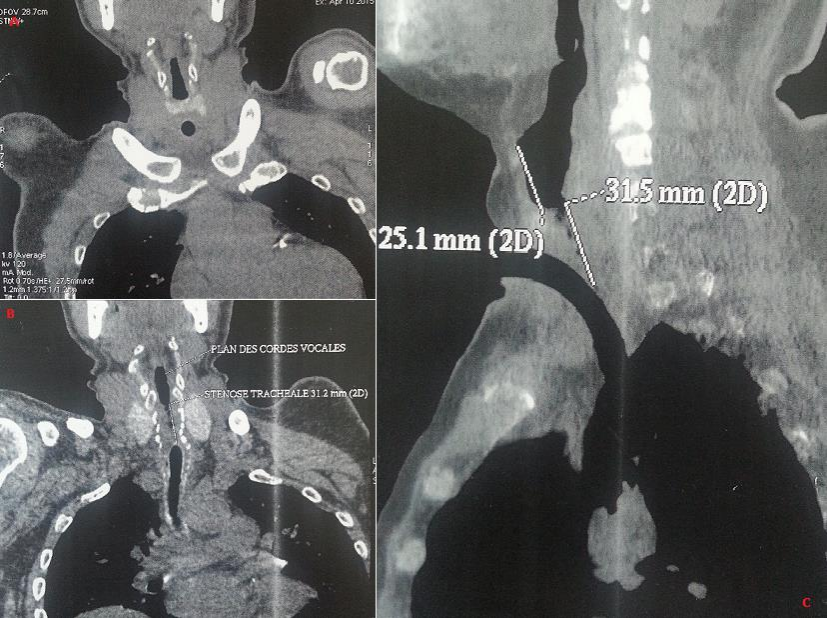
A) coupe scannographique frontale de la trachée: sténose trachéale au-dessus de l'orifice de trachéotomie; B) coupe scannographique frontale de la trachée: sténose trachéale étendue sur 3 cm; C) coupe scannographique sagittale de la trachée: sténose trachéale au-dessus de l'orifice de trachéotomie

